# Multiloss Function Based Deep Convolutional Neural Network for Segmentation of Retinal Vasculature into Arterioles and Venules

**DOI:** 10.1155/2019/4747230

**Published:** 2019-04-14

**Authors:** Sufian A. Badawi, Muhammad Moazam Fraz

**Affiliations:** ^1^School of Electrical Engineering and Computer Science, National University of Science and Technology, Sector H-12, Islamabad, Pakistan; ^2^The Alan Turing Institute, British Library, 96 Euston Road, London NW1 2DB, UK

## Abstract

The arterioles and venules (AV) classification of retinal vasculature is considered as the first step in the development of an automated system for analysing the vasculature biomarker association with disease prognosis. Most of the existing AV classification methods depend on the accurate segmentation of retinal blood vessels. Moreover, the unavailability of large-scale annotated data is a major hindrance in the application of deep learning techniques for AV classification. This paper presents an encoder-decoder based fully convolutional neural network for classification of retinal vasculature into arterioles and venules, without requiring the preliminary step of vessel segmentation. An optimized multiloss function is used to learn the pixel-wise and segment-wise retinal vessel labels. The proposed method is trained and evaluated on DRIVE, AVRDB, and a newly created AV classification dataset; and it attains 96%, 98%, and 97% accuracy, respectively. The new AV classification dataset is comprised of 700 annotated retinal images, which will offer the researchers a benchmark to compare their AV classification results.

## 1. Introduction

Arteries and veins are major components in the retinal fundus images, detecting early changes in those vessels are clear signs of detecting severity levels of the eye diseases [1, 2]. That can be diagnosed using the abnormalities in shape, size, and other morphological attributes of retinal vasculature [3]. Hence the study of tortuosity, appearance, shape, and further morphological attributes of human retinal blood vessels can be the important diagnostic indicator of several ophthalmic and system diseases that embraces diabetic retinopathy, hypertensive retinopathy, arteriolar narrowing, arteriosclerosis, and age-related macular degeneration [4].

The irregularities in retinal vasculature association by cardiovascular disease have been described in the studies [5–7]. The effect of systemic and ophthalmic disease on arterioles and venules is very much different. For instance, generalized arteriolar narrowing is one of the early signatures of hypertensive retinopathy. The decrease in Arteriole to Venule Ratio (AVR) is a famous predictor of stroke as well as other cardiovascular diseases in later life. Moreover, arteriovenous (AV) nicking is allied with long-term hypertension risk [5].

The advancement in retinal image acquisition and the availability of retinal fundus images made it possible to run large population-based screening programs to examine the early biomarkers of these diseases. Besides improving the diagnostic efficiency, the computerized retinal image analysis can help in reducing the workload of ophthalmologists. Therefore, an efficient algorithm for classification of retinal vasculature into the constituent venules and arterioles is an essential part of the automated diagnostic retinal image analysis system.

The arterioles/venules in the retinal images look much similar to each other with only very few known discriminating features [5]. The venules appear to be a little bit wider than the arterioles particularly in the place closer to the optic disc. The arterioles exhibit clearer and wider center light reflex as compared to the venules. The venules appear to be a bit darker in color than arterioles. Moreover, generally the arterioles do not cross other arterioles, and venules do not cross other venules within the retinal vasculature tree. The intra/interimage variability in color, contrast, and illumination are further added to existing challenges in developing an automated AV classification system. The widths, as well as the color of retinal vessels, change across their length as they have originated from optic disc and spread in the retina. The color change is due to the variability in the oxygenation level.

Deep learning [8] is gaining importance in the last few years due to the ability to efficiently solve complex nonlinear classification problems. The main advantage of deep learning is the automated feature learning from raw data. The convolutional neural network (CNN) [9] architectures have been utilized for a diversity of image classification and detection tasks with human level performance. The CNNs have been used to identify diabetic retinopathy in retinal images in recent Kaggle competition which gave very encouraging results. The promising results of CNN based architectures in retinal image analysis motivated the researchers to investigate the application of deep learning for pixel level classification and labelling.

Semantic segmentation of an image [10] alludes to the recognition of the image at the pixel level, and every pixel in the image will be classified or attributed to a given class. For the artery/vein classification problem, the target is to classify each pixel in the fundus image to one of the three classes, i.e., the artery, vein, or background.

In this paper, the authors have improved the work in [11], by proposing an optimized deep CNN based design, for classifying the retinal image pixels into arterioles and venules. The proposed technique can perform end-to-end training and classification of the vessels directly without the need for performing vessel segmentation as outlining the vessel centerlines [12] as proposed in different methods [13–16]. The optimization is achieved by (1) adding the segment-wise contextual judgment to the loss function that optimizes the vessel classification, (2) applying the optimized deep learning method on our newly prepared AV classification dataset to be published publicly after necessary paper work, and (3) improving the accuracy of the labels annotated as thin vessels. To the best of our knowledge, the deep learning-based pixel level semantic segmentation has been utilized for the first time in classifying retinal blood vessels into arterioles/venules. The proposed AV classification algorithm has a potential for replacing the AV classification module in QUARTZ retinal image analysis software tool [17], developed by our research group for quantification of retinal vessel morphology. QUARTZ aims to enable epidemiologists to analyse the association of retinal vessel morphometric properties [18] with the prognosis of various systemic/ophthalmic disease biomarkers [6].

This paper is arranged as follows: a brief evaluation of the related techniques is elucidated in Section 2. Sections 3 and 4 provide a comprehensive depiction of the proposed methodology and the new labelled dataset. In Section 5, the experimental outcomes are presented, followed by discussions and conclusion in Sections 6 and 7.

## 2. Related Work

A number of methods are available in the existing literature for classifying the retinal vasculature into arteries and veins. These methodologies might be classified into two major approaches: the feature-based and the graph-based approaches [19].

The feature-based approaches generate for each pixel a set of features that are used as input in an AV classification method. The initial step in most of these methods is the segmentation of the vessel's vasculature, followed by skeletonization of these vessels. The next step is crossovers and bifurcations identification.

The entire vasculature is partitioned into segments of the vessel by excluding the pixels at the bifurcation/crossover points from skeleton images. The features got captured from the segments, and then they are classified by a specific classifier to arteries or veins.

The graph-based methods usually model the vasculature tree as a graph planner. The contextual properties of the graph components are used to formalize the local decisions for each pixel whether it is arteriole or venule. Li [20] presented a Gaussian filter design to recognize the vessel's centerline reflex and utilized Least Mahalanobis Distance, but the accuracy of the classification is calculated at arteriole/ venule level, instead of pixel level.

Grisan [21] proposed segregating the retinal fundus image to four quadrants considering that each one of the segregated quadrants has a minimum of one artery/vein and subsequently performed fuzzy clustering method. Saez [22] and Vazquez [23] enhanced the quadrant based methodology, processed pixel-wise features from HSL and RGB color specifications, and used K-Mean method for AV classification. Kondarmann [24] proposed normalization for this background and then calculated the features of each pixel in the vessel centerline within a neighbourhood of 40 pixels and employed neural network for AV classification. Niemijar et al. [25] have processed a feature vector of the 27 dimensions for every pixel and characterized the vasculature sections utilizing a linear discriminating method for classification. Fraz [26] presented a feature vector at various levels (segment, pixel, and profile based) and performed the ensemble method for classifying the pixels. Relan [27] processed the feature vector from a circle-shaped neighbourhood around each pixel within a particular radius and classified the pixels using least square SVM. Xu [3] constructed a creative list of capabilities from the texture first- and second-order derivatives and forwarded it to KNN clustering. Rothaus et al. [2] and Dashtbuzorg et al. [28] have designed planner graph from vessel skeletons such that the crossovers and branches in the vascular tree are mapped to the graph nodes while the graph links represent the vessel segments. Rothaus et al. also have generated vessel graph, manually initialized some vessel segments, and propagated the vessel labels across the graph using a rule-based method. Dashtbozorg et al. [28] have merged the graph-based methodology with the supervised pixel classification method to achieve the classification at the pixel level. A 30-dimensional vector of color information-based features is computed for individual centerline pixel and then linear discriminant classifier was applied. These classification results are combined with graph labelling to improve the results. Global likelihood method is used by Estrada [29] to assign the links to the respective a/v labels.

The feature and graph-based methods may suffer in situations where the vessel vasculature tree is not segmented accurately. Moreover, these methods are deeply relying on manually designed features. Welikala et al. [30] have used for the first time deep learning for solving the problem of artery/vein classification. A convolutional neural network of six layers is used for learning the features in vasculature tree. The approach achieved significant results in terms of performance; however, it is also relying on accurate vessels segmentation of the retinal image. Sufian and Fraz [11] have proposed an end-to-end pixel level AV classification technique based on encoder-decoder based on fully convolutional neural network. This technique does not rely on the segmented vasculature; rather it learns and classifies the pixels directly from the image; however, the method is applied in a private dataset of 100 images only.

Normally the researchers go for selecting publicly available datasets or use their prepared ones that are suitable for their research purpose, whether it is vessel's segmentation, AV classification, or the analysis of the vessel's morphology. Public databases are very critical tools for the research community; they provide the needed data to develop and test new methods and enable comparisons with other approaches [31, 32].

A set of public databases are available publically for researchers, such as the de facto standard datasets DRIVE [31], STARE [33] databases, then CHASE-DB1 [12] for the segmentation of retinal blood vessels benchmarking, MESSIDOR [34], DRIONS-DB [35] for benchmarking and segmentation of optic nerve head [36], DIARETDB0 [37], DIARETDB1 [38], and HEI-MED [39] for benchmarking retinal lesions, like microaneurysms and exudates; several other databases are available such as REVIEW [40] and ONHSD [41]; however, for AV classification, the available datasets are very scarce.

Al Diri has published a set of (40) AV classification labels [35] as an extension to DRIVE dataset as this dataset is relatively small and may lead to inconsistent, contradictory comparison and produces subjective results among the approaches when used for deep learning approaches. The labelled gold standard scarcity leads the researchers to have no choice other than classifying a region of interest (ROI) in the retina as in [24, 25, 27, 42] or to benchmark their results using only selective data (distinguishable arteries and veins) as in [24] and in [43–45] to segment the exudates in the retinal background as in [46–48] to localise and segment the optic disk.

The lack of large labelled retinal fundus images dataset became an immediate necessity to be a benchmarking reference for the research community in AV classification, vessel segmentation, and related fields, especially for the introduced deep learning solutions that need a lot of data for more accurate learned models.

In this work, we have improved the deep learning network structure by introducing the contextual segment level judgment to the loss function, in addition to proposing a new labelled dataset composed of 700 labelled images for AV classification and vessel segmentation. The newly introduced large dataset is suitable for becoming a benchmark reference for AV classification and vessel segmentation purposes especially with the rise and success of the deep learning methods.

## 3. The Methodology

In this work, a multiloss function optimized deep encoder-decoder was designed based on entire convolutional neural system design for semantic segmentation of retinal vessels and simultaneously attaining the grouping of arteries and veins. The proposed optimized deep learning architecture takes motivation from SegNet [49] and achieved semantic classification of vessels to arteries and veins by assigning to each pixel of the retinal image a class label (arteriole, venule, or background pixel), without performing vessel segmentation separately, which normally had been a prerequisite step in the conventional computer vision artery/vein classification approaches.

The deep learning result that the authors have presented in [11] is optimized and improved in this work, by adding the segment-wise contextual judgment to the loss function that is targeting better optimization of vessel classification. The AV classification dataset is introduced as a large dataset that contains gold standard labels of AV classification and vessel segmentation. The dataset is built according to a systematic process as an extension and at the top of MESSIDOR and EPIC Norfolk. The optimized deep CNN is trained and evaluated on a large newly prepared AV classification dataset of 700 retinal images that helped to optimize the learned model and its results.

### 3.1. The Deep Network Architecture

The architecture consists of a sequence of encoder-decoder pairs which are used to create feature maps followed by pixel-wise classification.

**Figure 1:**
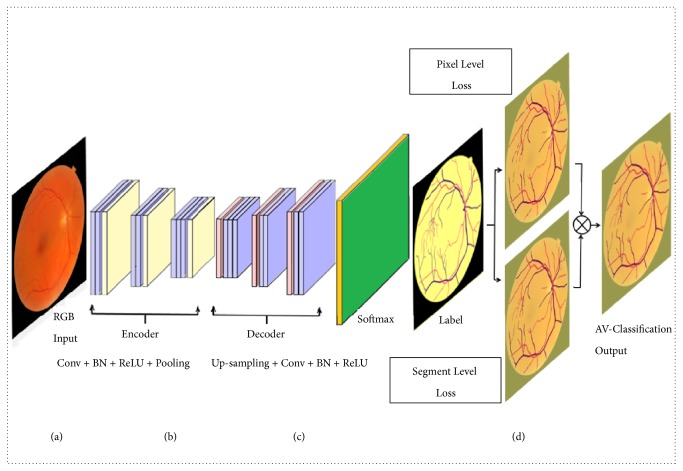
The network architecture: (a) the input retinal image; (b) the encoder section; (c) the decoder part; (d) the contextual segment-wise loss judgment enhancement.

**Figure 2:**
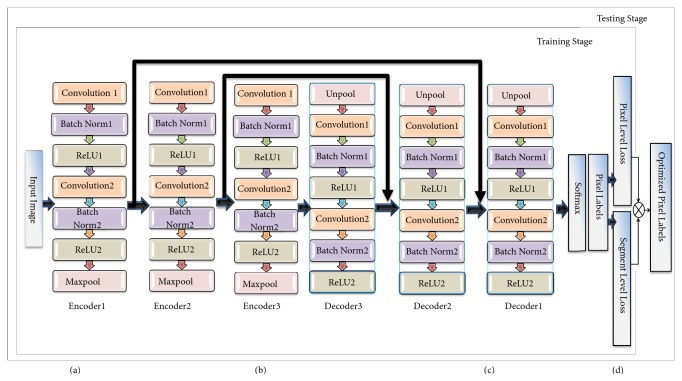
The network architecture; (a) the input block of the encoder part; (b) the complete network diagram; (c) the output block of decoder part; (d) multiloss function based optimized results.

Encoder-decoder architecture is demonstrated in Figures 1(b) and 1(c). The complete network comprises three layers of encoder-decoder blocks as shown in Figure 2. The input encoder block and output decoder block are presented in Figures 2(a), 2(b), and 2(c), respectively; Figures 1(d) and 2(d) illustrate the new enhancement that redirects the pixel-wise result to be adjusted by segment-wise majority vote judgment that optimizes the loss function. Without any fully connected layers, the network is consisting of convolutional layers which are typically established at the end of the traditional CNN. The encoder-decoder based fully convolutional neural network considers the arbitrary size input and given output accordingly sized. The feature learning and inference are achieved as a whole-image-at-a-time basis by back-propagation and dense feedforward computation.

The encoder part of the network takes an input image and generates a high-dimensional feature vector by learning the features at multiple abstractions and aggregating the features at multiple levels. The decoder part of the network takes a high-dimensional feature vector and generates a semantic segmentation mask. The building blocks of the network are convolutional layers, downsampling, and upsampling. The learning is performed within subsampled layers using stride convolutions and max pooling. The upsampling layers in the network enable pixel-wise prediction by applying unpooling and deconvolutions.

### 3.2. The Multiloss Function

The pixel-wise loss is the traditional way to compare the results of the segmented image with the ground truth label. It is achieved by comparing the pixel from the segmented image to the same pixel in the ground truth and building the confusion matrix and performing the calculation of desired performance measures.

In arterioles and venules (AV) classification problem the pixel-wise loss calculation is having an issue due to the severe inconsistent thickness of the resulting thin vessels; the thin vessels in the labelled image are defined as the vessels that are less than four-pixel width. The impact of this issue on the results is high since the thin arteries and veins in the retina represent 77% of the retinal vessels, and the inconsistent thickness of those thin arteries and veins deviate the results as well as causing wrong judgment for some pixels that belong to arteries as vein pixels and vice versa.

To overcome this problem, we have improved our deep learning published approach used in [11] for AV classification by including the segment-wise loss to the model that increases the accurate results and reduces the bias in sensitivity and specificity inspired from the skeletal method in [50, 51]. New steps are added to optimize the AV classification results. Instead of using a pixel-to-pixel similarity measure, each skeleton segment in the reference skeleton map is assigned adaptively with the color that achieves optimal loss calculation. The result will be determined by selecting the class color generated from the SoftMax function or the color of the class achieved by the majority of the pixels in the skeleton segment. The pixels that exist in the same targeted segment which has the skeleton map got the color judgment of the segment skeleton map color. This enhanced loss calculation hence improves the results and fixes the few artery segment pixels that are wrongly judged as vein pixels, and conversely, it fixes the few vein pixels that are judged as arteries in deep learning.

### 3.3. Learning Details

The methodology is evaluated on the AV classification dataset that contains 700 images detailed in Section 4, such that 90% of the images are used for training, and 10% of the images are used for testing.

**Figure 3:**
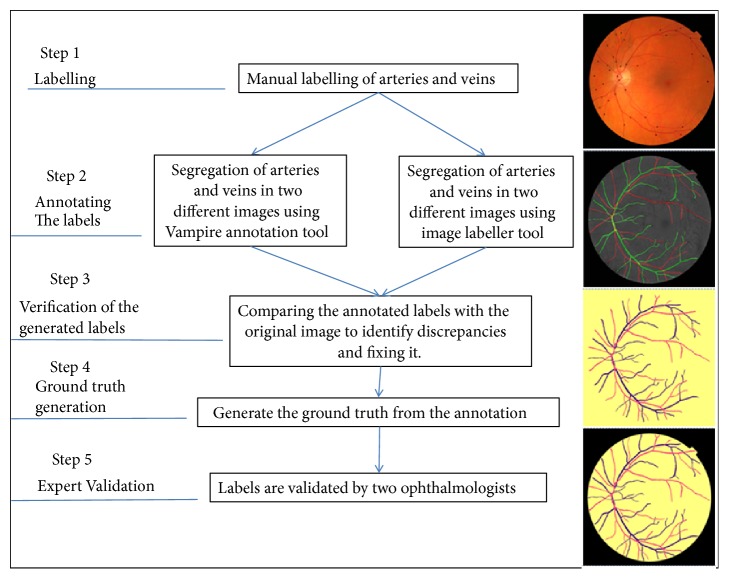
AV classification labels, as well as vessel segmentation, labels creation methodology.

**Figure 4:**
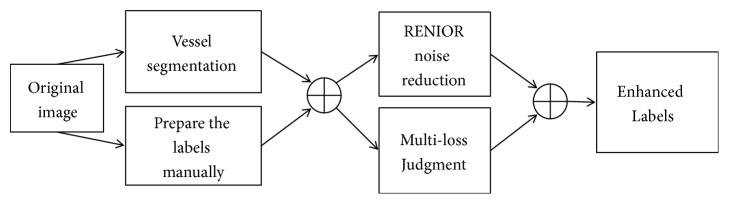
Thickness inconsistency penalization using segment level loss for the manually prepared labels.

**Figure 5:**
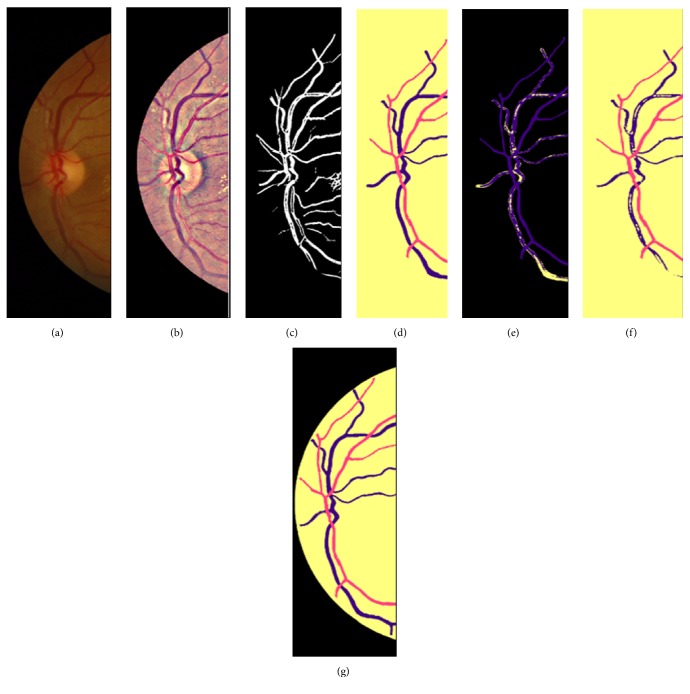
Vessel thickness inconstancy improvement in the manually prepared label: (a) original image; (b) local contrast normalization; (c) vessel segmentation; (d) manual labelling; (e) visualizing the thickness inconsistency between (c) and (d); (f) results of adjusting results of (c) on (d); (g) optimized labels image after applying enhancement procedure in Figure 4.

**Figure 6:**
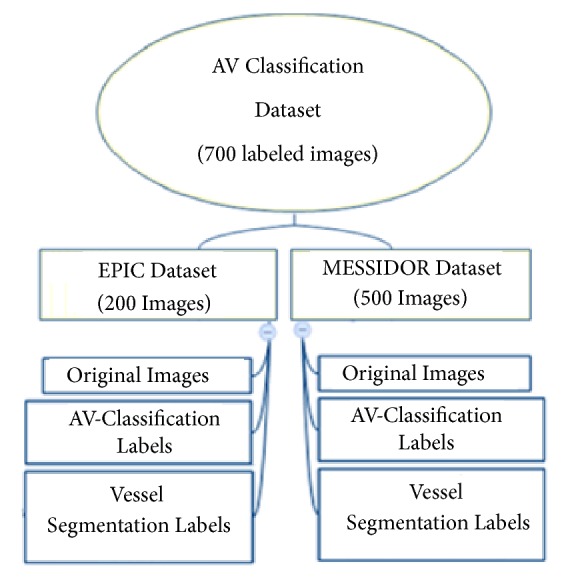
The proposed AV classification dataset for deep learning that contains AV classification labels as well as vessel segmentation.

**Figure 7:**
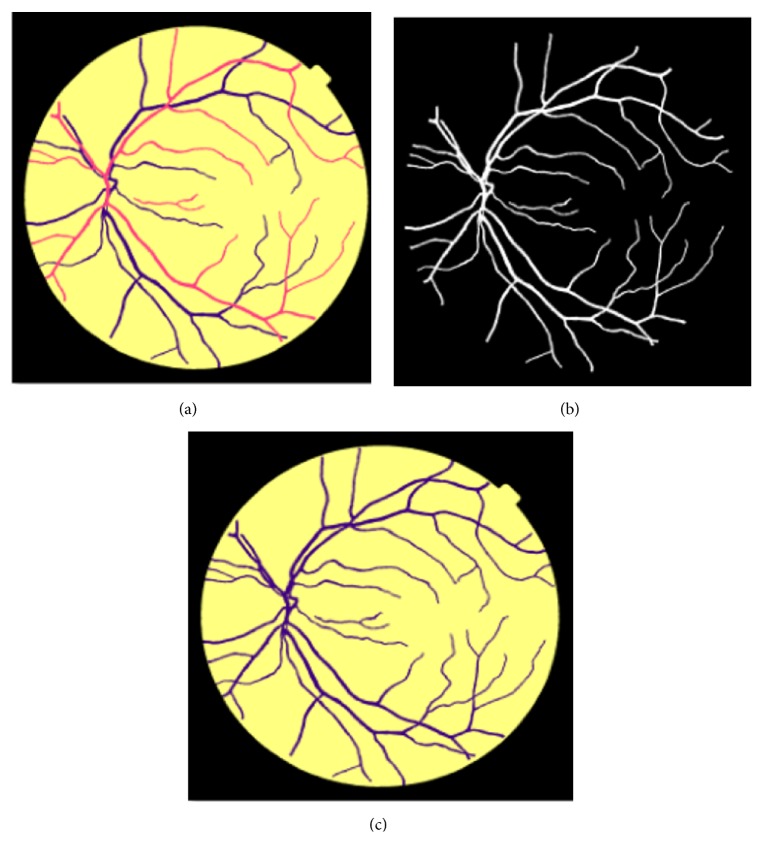
AV classification dataset sample labels: (a) AV classification label; (b) black and white vessel segmentation label; (c) colored vessel segmentation label.

**Figure 8:**
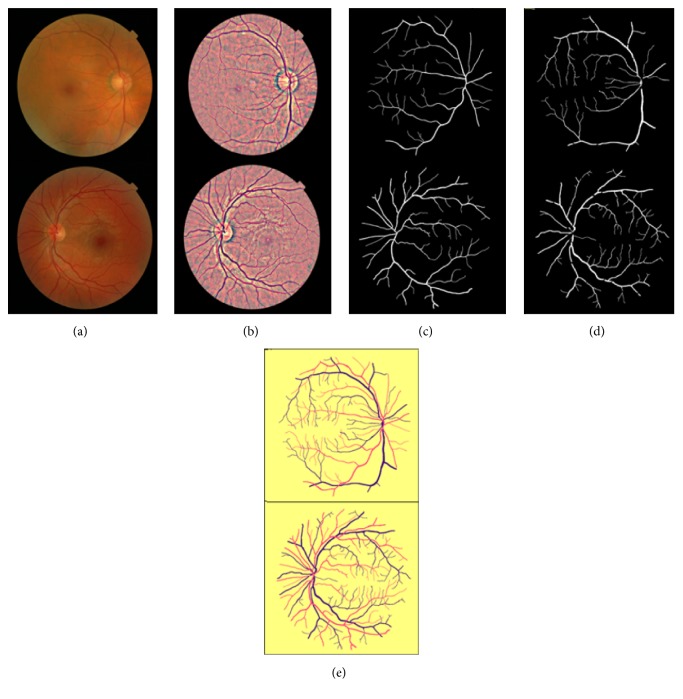
Creating AV classification semantic labels for MESSIDOR and EPIC datasets, with the following stages: (a) original; (b) preprocessed; (c) arteries; (d) veins; (e) AV classification semantic labels images.

The available pertained models which include AlexNet, VGG, and ResNet are trained on PASCAL VOC [52] or [53]. These datasets are very much different from that of retinal images. Therefore, the pretrained weights are not used. The stochastic gradient descent (SGD) is used to train all the network. The learning rate fixed at 0.1 and a mini-batch of 12 images are used for training. The used approach is adding an extra step for calculating the multiloss function judgment for each pixel and taking the optimal loss value as shown in Figure 1 and (1)-(8). The newly prepared AV classification dataset is used to measure and benchmark the deep learning encoder/decoder performance; then it is tested on DRIVE and AVRDB [54] public datasets.

The challenge is to achieve the segmentation of the three classes (arteries, veins, and background). The RGB images resolution is 2000×2002. Normalization of the local contrast [55] is performed for the original images. Following SegNet semantic segmentation in [49] and using median class frequency balancing in training introduced in [49], the weights of the decoder were initialized using “MSRA” technique for the weight initialization. We followed each deep learning layer by a ReLU nonlinear unit. The learning rate is set to 0.1, and stochastic gradient descent (SGD) is used to train all the variants where each epoch starts after shuffling the training set. Each mini-batch (4 mages) is then fetched to ensure the one time use of each image. The objective function used for the training is the network cross-entropy [56]. The loss is summed up over all the pixels in a mini-batch. The training is continued till convergence and reaching the optimal training loss. The model with the highest performance results on the validation dataset is selected.

To calculate the segment level loss, we start with the binary image and generate the vessels vasculature tree using [54]; then we proceed in skeletonization using morphological processing to generate entire vessels skeleton Js; then we identify the bifurcation and intersection points that are then omitted to generate the set of vessel segments. S={Sj : where J = 1,  N}, and N is the number for segments in the retinal image, considering that we have used a hyperparameter Maxlength to represent the maximum length for the segment that is defined as the summation of all deviations of all the segment pixels from the maximum diameter (Tp).

(1) MaxLength=∑P∈SjTp−TVjSj

(2) Js=Entire  skeleton  segment=∑1NSj

(3) Vj=The  vessel  pixels  in  segment  contaning  the  skeleton  segment  Sj

(4) TVj=VjSj=Count  of  pixels  in  VjLength  of  Sj

The same is done for the predicted image results where equivalently we get J'_s_, S'_j_, V'_j_, and T_V_j__′ to optimize the vessel color; every vessel segment Vj in Js is allocated to a specific range Rj that is defined as the minimum radius that guarantees the maximum overlap between Vj and V′j to discover corresponding pixels color in the predicted image to perform the judgment. By manipulating the predicted image during the process of training, we produce a twofold guide by performing a threshold of 0.5 (to be specific every pixel in the predicted image will be classified as artery or vein or by checking its probability value with the threshold 0.5).

Then, for every vessel segment V_j_ in J, the color of all the pixels in the predicted image located within the searching range R_j_ forms a vessel segment denoted as Vs′_j_. The thickness inconsistency of the vessel or the pixel color inconsistency between Vs′_j_ and V_j_ is measured by defining the mismatch ratio (MisR) as

(5) $$\begin{array}{c} MisR\left(\;{VS^{\prime }}_{j},V_{j}\;\right)=\frac{\left|T_{{Vs}_{j}}-T_{V_{j}}\right|}{T_{V_{j}}} \end{array}$$

To measure the segment level loss, we construct a weight matrix defined as

(6) wp=1+MisRVs′j,Vjif  p∈Vs′j,  j=1,2,3,…,N1other  wise

where each pixel (p) is assigned a weight w_p_ and used to calculate the loss value of predicting the color of the pixel P_predicted_ in the predicted image and is compared to the same pixel P_label_ in the original label we calculated a weighted loss:

(7) $$\begin{array}{c} P_{loss}=\left|\;P_{predicted}-P_{label}\;\right|.w_{p} \end{array}$$

This is compared with the probability value of the pixel-wise loss p to calculate the segment-loss that builds the segment level loss judgment and adaptively builds the matrix for artery and vein loss probabilities. P_*artery*_ represents the artery level loss while P_*vein*_ represents the vein level loss where both contribute to creating the multiloss function judgment for the vessel pixel as in (8); it leads to assigning the pixel class color based on the segment level loss if the pixel-wise loss shows prediction deviation in some of the segment pixels.

(8) $$\begin{array}{c} Multi\text{-}loss\;\;Judgment=\max\left\{\;1\text{-}P_{artery},1\text{-}P_{vein}\;\right\} \end{array}$$

## 4. AV Classification Dataset Preparation

A plan is prepared to generate a new dataset of 700 AV classification labels for retinal fundus images for AV classification supervised problems and another set of 700 AV-vessel segmentation labels for retinal fundus images for vessel segmentation problem. The methodology follows the guidelines approach and specified methodology (Figure 3).

### 4.1. Vessel Labelling Approach

The physiological characteristics of arteries and veins in the retina give us a clue of confidently differentiating arteries from veins manually. The vessel's annotation condition depends on the characteristics and the morphological features of the vascular system of the retina [57]. Below is the rules list, used through the manual labelling systematic process.

1. The arteries are brighter than the veins, usually thinner than neighbouring veins, and have their central reflex usually brighter/wider than veins [24].
2. When a vessel divides into two branch segments, the width of the parent vessel is greater than the width of any of its branches, and the angle between the parent vessel and the branch's segment is not greater than 90 degrees [58].
3. Parallel vessel segments are typically contrary in type [24].
4. The vessels, crossing each other, must be from the opposite class.

### 4.2. Labelling Methodology

For vessel labelling, we measured the original color images, the green channel of the images, and the grey-scaled preprocessed version images. The vessel-type labelled set for the vessels is created according to the process shown in Figure 3. The vessel's labelling is performed in a systematic process, which starts with manual marking, according to a set of physiological characteristics of the retina. Then annotation is performed using image labeller using a preprocessed version of the original images, by two computer vision specialists and an ophthalmologist.

The process of validation includes counter validation by specialists in computer vision and reviews verification by an ophthalmologist for the class of the vessels. We started in the first stage by the manual marking of the vessels on a preprocessed image. The preprocessing aim is to increase the discrimination between arteries, veins, and the background color to have an enhanced version of the image. The preprocessing is achieved by applying morphological processing, normalization, and intensity averaging for each RGB layer and then mapping the intensity values in the grey scale of each layer to new values, by saturating the bottom 2% and the top 3% of all pixel values. This operation increased the contrast of the output preprocessed image. The vessels labelling methodology is completed in the following steps:

1. The line operator filter method is utilized to attain an image vessels enhanced version. The preprocessed image is used for marking artery as “a” and vein as “v”.
2. The vessels are manually labelled by an expert using the artery vein known distinguishing features explained in Section 4.1; then the vessels are annotated by two experts using image labeller application available with Matlab as well as the VAMPIRE vessel segmentation tool [59].
3. Validation activity is then performed by observers who collaborate during the labelling process to review and finalize the annotation type of entire segments of the vessel. The label is finally generated from the reviewed annotations.
4. The next step in the labels preparation is the generation of the AV classification labels as well as the vessel segmentation labels from the original images and the annotations.

Two ophthalmologists verify all the labels at St. Georges University of London, UK. The validation step ensures the complete agreement between the reviewers and annotators; in case there is a comment, the reviewers report it to the annotators for fixing and resending again till the complete agreement is achieved; this also offers reliable AV classification labels dataset for the research scholars in the field.

### 4.3. Labels Enhancement

The manual labelling and annotation of the vessels are achieved by noticing the continuity of the vessel's type throughout the vessels branching, referring to the physiological characteristics of retinal vessels, and the observed attributes of the vessels following the labelling method detailed in the previous section.

After completing the manual preparation of the labels in Figure 3, we have applied the following steps to enhance the prepared labels:

1. Extracting the vessel tree of the original images in the black and white image [60]
2. Combining the vessel tree results above with the manually prepared label to enhance the labels edges
3. Morphological processing to close the holes in the segmented vessels
4. Noise removal [61] to clean the background
5. Performing the multiloss judgment for each generated label to enhance the thickness inconsistency of the pixels beyond the segmented edges as illustrated in and in Figures 4 and 5

Figure 5(e) visualizes the vessel thickness inconstancies between the vessel segmentation and the manual annotation enhancement to improve the labels; some images are suffering from poor contrast, illumination, or some pathologies. Hence it affects the manual annotation process that impacts the annotated vessel widths when compared to normal segmented width.

## 5. Experimental Evaluation

### 5.1. Materials

The methodology is evaluated on the new AV classification dataset that is prepared to be suitable for deep learning purposes especially for artery/vein classification and vessel segmentation as it contains labels for both types of problems.

The dataset is created by preparing three new types of labels for each image; the first type is created for artery/vein classification purposes while the second and third are created for vessel segmentation purposes. The original images are a subset of images from EPIC and MESIDOR datasets. Seven hundred labels are created for each type, two hundred of them are created for images from EPIC dataset, and the five hundred are created for MESSIDOR dataset. The colored AV classification labels are used from created AV classification dataset to implement the deep learning optimized method.

#### 5.1.1. The AV Classification Dataset

The labels creation methodology is implemented to generate three types of labels (colored AV classification labels, colored vessel segmentation labels, and black and white vessel segmentation labels).

The images and their labels are of 2002 X 2000-pixel sizes and the first experiment was done and AV classification deep learning problem was also handled in this paper. The high-level dataset hierarchy is illustrated in Figure 6 that contains two subfolders; one for EPIC 200 images and the other for MESSIDOR 500 images, and each image appears in the subfolders (original, AV classification labels, and vessel segmentation labels containing subfolder for the colored labels and another for the black and white label types).

The AV classification colored label Figure 7(a) consists of arteries (red), veins (blue), and background (yellow). The vessel segmentation labels are of two formats. The first format is in Figure 7(b) that is a standard black and white binary image.

The AV classification colored label Figure 7(a) consists of arteries (red), veins (blue), and background (yellow). The vessel segmentation labels are of two formats. The first format is in Figure 7(b) that is a standard black and white binary image. The second vessel segmentation format is in Figure 7(c) that is a colored image where vessels are in “blue” color, and the background is in “yellow” color.

For each image two types of labels are prepared: one for AV classification (check Figure 8 for the prepared MESSIDOR and EPIC AV classification labels) and the other for vessel segmentation with image resolution 2002x2000.

#### 5.1.2. DRIVE Dataset

DRIVE [31] dataset is a consistent set which consists of fundus images utilized for benchmarking of classification and vessel segmentation effectiveness. These images were taken in the Netherland for making the screening the diabetic retinopathy. Within the age group of 25-90 years, data of a total number of 400 diabetic subjects is composed. All the images were compacted with JPEG. For capturing these images, Canon CR5 3CCD nonmydriatic camera is used. Each image's resolution is 768 by 584 pixels having 8 bits per pixel. In each training and test dataset, a set of 20 images are presented. The dataset comprises manually organized ground truth images and masks which are made by two specialists.

#### 5.1.3. AVRDB Dataset

AVRDB database [54] is developed to be publicly available for hypertensive retinopathy detection. It contains 100 images that are captured via TOPCON TRC-NW8. The dataset annotated labels are prepared in coordination with AFIO, Pakistan expert ophthalmologists. The vasculature tree is classified into venular and arteriolar. The 100 images' size is 1504×1000, and it contains retinal veins, arteries, and AVR, with the vascular, arteriole, and venule branching and mapping of artery and vein network on original fundus image.

### 5.2. Performance Measures

The performance measures defined in Table 1 are implemented to quantify the performance of the proposed method.

### 5.3. AV Classification Results

#### 5.3.1. Optimized Multiloss Function Judgment

The proposed improved segment-wise judgment has enhanced the AV classification pixel-wise results. The pixel-wise judgment is adjusted by comparing it with the contextual color of the adjacent pixels in the segment (see Table 2).

Table 4 shows that the performance is higher when applying the step of multiloss judgment in the FCN method. The proposed method is tested in the AV classification dataset and showed 97.03% while by testing it with the datasets of DRIVE and AVRDB it showed 96.07% and 98.13%, respectively.

#### 5.3.2. Visualization of Results

We have run the experiments twice on the 700 prepared images. The first time is with the FCN and then the experiment is repeated using the improvement of segment-wise loss calculation. This approach enhanced the results as shown in Table 3.

**Figure 9:**
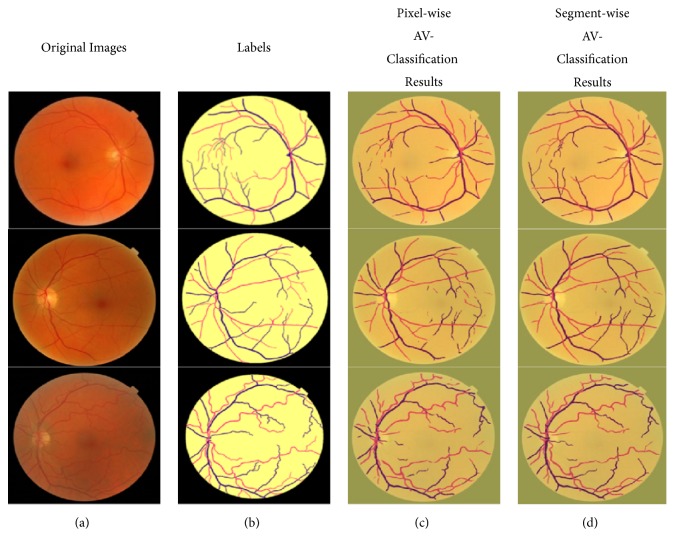
AV classification results on newly proposed AV classification dataset: (a) original images; (b) labels; (c) pixel-wise deep learning results; (d) multiloss function deep learning results.

**Figure 10:**
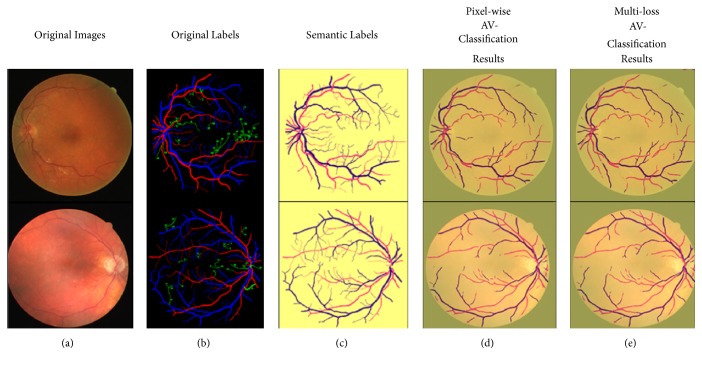
AV classification on DRIVE dataset: (a) original images [31]; (b) original labels [35]; (c) semantic labels; (d) pixel-wise deep learning results; (e) multiloss function deep learning results.

**Figure 11:**
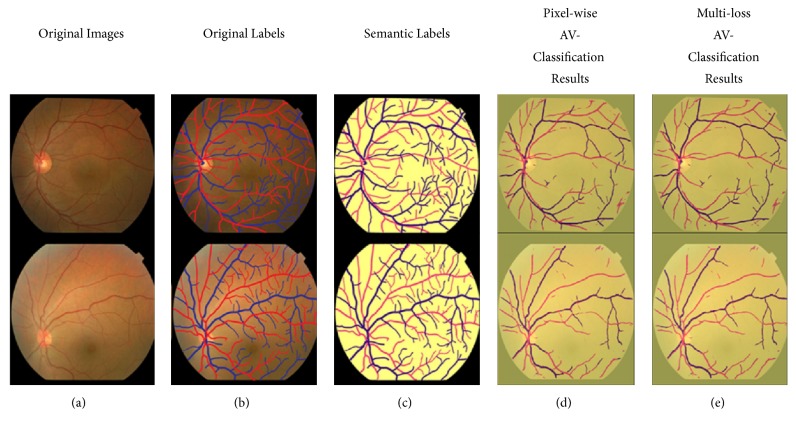
AV classification results on AVRDB dataset: (a) original images [54]; (b) original labels; (c) semantic labels; (d) pixel-wise deep learning results; (e) multiloss function deep learning results.

Table 4 and the consequent output results in Figures 9–11 are showing the performance output images before and after adding the improvement of segment-wise contextual judgment for AV classification results.

Figure 9 shows the classification outcomes of the proposed methodology on AV classification dataset. The first column corresponds to the retinal image. The second column belongs to the ground truth. The third column represents the AV classification performance results when the pixel classification is generated. And the fourth column is the output after performing the segment-wise optimization for the generated pixel-wise results.

The sample images are from the newly prepared AV classification dataset. The semantic representation of the label colors is as follows: the background is marked with yellow color, and the arterioles and venules are marked with red and blue color, respectively.

Figures 10 and 11 show the AV classification results on DRIVE and AVRDB datasets. The first column shows the retinal image. The second column illustrates the ground truths and the third column demonstrates the semantic segmentation label. The fourth column represents the AV classification results where the pixel classification is generated, and the fifth column is the output after performing the segment-wise optimization for the generated pixel-wise results. The semantic representation of the label colors is as follows: the background is marked with yellow color, and the arterioles and venules are marked with red and blue color, respectively.

**Figure 12:**
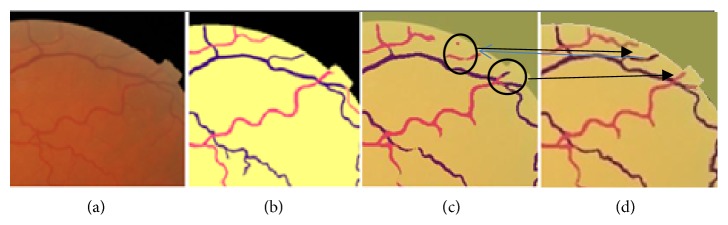
Example of how the segment-wise loss judgment enhanced the segmentation results. The images are from (a) original image, (b) label, (c) pixel-wise deep learning loss, and (d) multiloss function deep learning results. The two circles in Figure 12(c) show the wrong judgment that is corrected by multiloss function improvement in our proposed method.

Figures 9–11 are showing the benefits achieved from applying multiloss function. Example of how the segment-wise loss judgment enhanced the segmentation results is shown in Figure 12.

#### 5.3.3. Comparison with Other Methods

The proposed approach's performance is compared along with the previously established methodologies in AV classification. The proposed optimized method has achieved better results in deep learning of classifying arteries from veins in the retinal images. The comparison of the algorithm accuracy with the previously published algorithms is shown in Table 4. We notice a clear observation in Table 4 about the use of several private datasets by several researchers, and the rest have used DRIVE dataset that contains only 40 images. Moreover, most of the introduced AV classification methods have used pixel-to-pixel similarity check, to measure the performance of their approaches and to compare it with previous works in the field; such metric is affected by the observer-to-observer vitiations in the vessel location and thickness; hence it adds some issues to benchmarking or comparison judgment. For example, (1) they are different in the used dataset, (2) the methods of measuring the performance are not the same, (3) the datasets used are relatively small, and finally, (4) the sizes of the used datasets are insufficient, especially for deep learning approaches.

Hence we have proposed using new large dataset and applying the segment-wise judgment to achieve the standard comparison between the methods and also to improve the reliability, robustness, and credibility of the proposed solutions. The proposed method achieved 97.03% accuracy on the AV classification dataset and showed 95.5% and 98.5% accuracy on DRIVE and AVRDB datasets, respectively.

## 6. Discussion

Most of the introduced AV classification methods have used pixel-to-pixel similarity check to measure the performance of their methods in comparison with the previous works in the field. This method suffers from observer-to-observer variations in the vessel location and thickness and the use of small/private datasets that are not suitable for deep learning approaches.

The AV classification researchers community lacks the existence and the availability of the public large AV classification image set to assess the performance of a proposed method in terms of reliability and robustness. This technique improved FCN deep learning results from 93.5% in [11] to 97.03%; in addition, it is used in enhancing the new AV classification labels (Figure 8). In this work, the newly introduced and used AV classification dataset contains standard labels created at the top of MESSIDOR and EPIC datasets, and the introduced and used the dataset in deep learning has reported a 97% accuracy for AV classification when compared to the previous works. It is achieved by applying the deep learning on a larger dataset and enhancing the result through the application of the segment-wise loss judgment. Therefore, the results are more credible. The vessels thickness inconsistency in the AV classification dataset labels is penalized using the contextual segment level loss judgment (see Section 4.3: Labels Enhancement).

The AV classification dataset that is used in this work will be released publicly for the MESSIDOR images. The research community can use this dataset in their experiments in the problems of AV classification or vessel segmentation. The AV classification or vessel segmentation labels are available in black and white or semantic colored format.

## 7. Conclusion

The medical imaging research field lacks the availability of a large labelled retinal images dataset for assessing the AV classification method and comparing it with other researchers' methods, by benchmarking with central reference large dataset for medical images.

In this paper, an improved result is introduced for a novel encoder-decoder based fully convolutional deep network for robust AV classification of retinal blood vessels. An optimized, proposed segment-wise judgment technique for improving the AV classification results is presented. The use of multiloss function and utilizing the segment level contextual judgment have enhanced the AV classification accuracy results from 93.5% to 97% in this work. A new AV classification dataset is prepared as an extension to MESSIDOR and EPIC Norfolk datasets. It contains 700 fundus images for AV classification and the same and vessel segmentation.

This new AV classification/vessel segmentation labelled retinal images database and the proposed multiloss function judgment technique allow more accurate evaluation and benchmarking with the previous methods in terms of reliability, robustness, and performance. The three types of labels in the AV classification dataset of MESSIDOR images will be released publicly so that the research community can use it in their experiments. The AV classification database will be available at http://vision.seecs.edu.pk/dlav/ or by emailing the authors; we welcome opinions about their experience of using this dataset to keep the AV classification dataset updated.

In future, we aim to extend this methodology to be used in place of the current AV classification module in the QUARTZ software, which is developed by the research group for automated quantification of the retinal vessel morphometry, with the aim of studying associations between vessel change and systemic/ophthalmic disease prognosis. Furthermore, we aim to use the proposed methodology as a preliminary step in the development of the modules in QUARTZ for the identification of venules beading and the measurement of arteriovenous nicking.

## Figures and Tables

**Table 1:** Performance measures applied in artery/vein classification.

Metric	Description
Mean Accuracy	the ratio of the correctly classified pixels averaged over the classes
Global accuracy	It measures the ratio of pixels accurately classified in the dataset. Global Accuracy = (TP + TN)/(TP + FP + FN + TN)
Mean Intersection over Union (mIoU)	The similarity comparison between the class resulting from the prediction and the actual ground truth class
Weighted Intersection over Union (wIoU)	Performs IoU average weighted by the number of pixels in every class
Mean BF Score	This performance metric measures how accurate the boundaries of the predicted objects match the boundaries of the ground truth

*∗* TP, TN, FP, and FN are abbreviations of true positive, true negative, false positive, and false negative, respectively.

**Table 2:** Optimized multiloss function judgment.

ROI	Total# of Pixels	Label class	Total# of Red Pixels	Total# of Blue Pixels	Artery Probability	Vein Probability	Maximum Probability	Multi- Loss Judgment
Seg1	200	Artery	120	80	0.600	0.400	Red	Artery
Seg2	500	Vein	400	100	0.800	0.200	Blue	Vein
Seg3	700	Vein	100	600	0.100	0.900	Blue	Vein
Seg4	457	Vein	46	411	0.100	0.900	Blue	Vein
Seg5	458	Artery	320	137	0.700	0.300	Red	Artery
Seg6	459	Vein	0	459	0.000	1.000	Blue	Vein
Seg7	460	Vein	184	276	0.400	0.600	Blue	Vein
Seg8	320	Vein	43	277	0.134	0.867	Blue	Vein
Seg9	230	Artery	146	84	0.634	0.367	Red	Artery
Seg10	543	Artery	326	217	0.600	0.400	Red	Artery
Seg11	544	Artery	508	36	0.934	0.067	Red	Artery
Seg12	545	Artery	529	18	0.967	0.034	Red	Artery

**Table 3:** Quantitative performance measures of AV classification.

Dataset		Global Accuracy	Mean Accuracy	Mean IoU	Weighted IoU	Mean BF Score
AV-Classification	Pixel Level	0.9652	0.8228	0.7905	0.9457	0.8201
	Multi-level (Segment/Pixel)	0.9703	0.8739	0.8506	0.9568	0.8313

DRIVE	Pixel Level	0.9492	0.6823	0.55904	0.9207	0.74312
	Multi-level (Segment/Pixel)	0.9607	0.6913	0.5666	0.9378	0.75547

AVRDB	Pixel Level	0.9650	0.7009	0.4322	0.8562	0.70134
	Multi-level (Segment/Pixel)	0.9813	0.7109	0.4375	0.8602	0.70216

**Table 4:** Performance comparison with different AV-classification methods.

S. No	Algorithm	Pixel Level Accuracy	Segment Level Accuracy	Used Dataset
1	Grisan[21]	-	87.58%	Private
2	Kondarmann [24]	95.32%	-	Private
3	Niemeijer [25]	93.5%	-	Private
4	Vazquez [23]	87.68%	-	VICAR
5	Fraz [26]	83.27%	-	Private
6	Relan [27]	-	89.4%	DRIVE
7	Xu [3]	92.3%	-	DRIVE
8	Dashtbuzorg [28]	87.4%	-	DRIVE
9	Estrada [29]	91.97%	93.5%	DRIVE
10	Welikala [30]	91.97%	91.27%	DRIVE
11	Sufian and Fraz [11]	93.5%	-	Private
12	Proposed Methodology	**94.9%**	**96.07%**	DRIVE
		**96.5%**	**98.13%**	AVRDB
		**96.5%**	**97.03%**	AV-classification

## Data Availability

The data used to support the findings of this study are available from the corresponding author upon request.

## References

[1] Welikala R. A., Fraz M. M., Dehmeshki J. (2015). Genetic algorithm based feature selection combined with dual classification for the automated detection of proliferative diabetic retinopathy. *Computerized Medical Imaging and Graphics*.

[2] Rothaus K., Jiang X., Rhiem P. (2009). Separation of the retinal vascular graph in arteries and veins based upon structural knowledge. *Image and Vision Computing*.

[3] Xu X., Ding W., Abràmoff M. D., Cao R. (2017). An improved arteriovenous classification method for the early diagnostics of various diseases in retinal image. *Computer Methods and Programs in Biomedicine*.

[4] Kanski J. J., Bowling B. (2015). *Clinical Ophthalmology: A Systematic Approach*.

[5] Wong T. Y., Klein R., Sharrett A. R. (2002). Retinal arteriolar narrowing and risk of coronary heart disease in men and women. *Journal of the American Medical Association*.

[6] Owen C. G., Rudnicka A. R., Welikala R. A. (2019). Retinal vasculometry associations with cardiometabolic risk factors in the european prospective investigation of cancer—norfolk study. *Ophthalmology*.

[7] Welikala R. A., Fraz M. M., Foster P. J. (2016). Automated retinal image quality assessment on the UK Biobank dataset for epidemiological studies. *Computers in Biology and Medicine*.

[8] LeCun Y., Bengio Y., Hinton G. (2015). Deep learning. *Nature*.

[9] Simonyan K., Zisserman A. Very deep convolutional networks for large-scale image recognition. https://arxiv.org/abs/1409.1556.

[10] Shuhan C., Ben W., Jindong L., Xuelong H. Semantic image segmentation using region-based object detector.

[11] Badawi S. A., Fraz M. M. Arterioles and venules classification in retinal images using fully convolutional deep neural network.

[12] Fraz M. M., Barman S. A., Remagnino P. (2012). An approach to localize the retinal blood vessels using bit planes and centerline detection. *Computer Methods and Programs in Biomedicine*.

[13] Fraz M. M., Rudnicka A. R., Owen C. G., Barman S. A. (2014). Delineation of blood vessels in pediatric retinal images using decision trees-based ensemble classification. *International Journal for Computer Assisted Radiology and Surgery*.

[14] Fraz M. M., Remagnino P., Hoppe A. (2012). Blood vessel segmentation methodologies in retinal images—a survey. *Computer Methods and Programs in Biomedicine*.

[15] Fraz M. M., Remagnino P., Hoppe A. (2012). An ensemble classification-based approach applied to retinal blood vessel segmentation. *IEEE Transactions on Biomedical Engineering*.

[16] M Fraz M., Basit A., Barman S. A. (2013). Application of morphological bit planes in retinal blood vessel extraction. *Journal of Digital Imaging*.

[17] Fraz M. M., Welikala R. A., Rudnicka A. R., Owen C. G., Strachan D. P., Barman S. A. (2015). QUARTZ: Quantitative analysis of retinal vessel topology and size - An automated system for quantification of retinal vessels morphology. *Expert Systems with Applications*.

[18] Fraz M. M., Remagnino P., Hoppe A. (2013). Quantification of blood vessel calibre in retinal images of multi-ethnic school children using a model based approach. *Computerized Medical Imaging and Graphics*.

[19] Miri M., Amini Z., Rabbani H., Kafieh R. (2017). A comprehensive study of retinal vessel classification methods in fundus images. *Journal of Medical Signals and Sensors*.

[20] Li H., Hsu W., Lee M., Wang H. A piecewise Gaussian model for profiling and differentiating retinal vessels.

[21] Grisan E., Ruggeri A. A divide et impera strategy for automatic classification of retinal vessels into arteries and veins.

[22] Saez M., González-Vázquez S., González-Penedo M. (2012). Development of an automated system to classify retinal vessels into arteries and veins. *Computer Methods and Programs in Biomedicine*.

[23] Vázquez S. G., Cancela B., Barreira N. (2013). Improving retinal artery and vein classification by means of a minimal path approach. *Machine Vision and Applications*.

[24] Kondermann D. K. C., Yan M. (2007). Blood vessel classification into arteries and veins in retinal images. *Medical Imaging*.

[25] Niemeijer M., Xu X., Dumitrescu A. V. (2011). Automated measurement of the arteriolar-to-venular width ratio in digital color fundus photographs. *IEEE Transactions on Medical Imaging*.

[26] Fraz M. M., Rudnicka A. R., Owen C. G., Strachan D. P., Barman S. A. Automated arteriole and venule recognition in retinal images using ensemble classification.

[27] Relan D., MacGillivray T., Ballerini L., Trucco E. Automatic retinal vessel classification using a Least Square-Support Vector Machine in VAMPIRE.

[28] Dashtbozorg B., Mendonça A. M., Campilho A. (2014). An automatic graph-based approach for artery/vein classification in retinal images. *IEEE Transactions on Image Processing*.

[29] Estrada R., Allingham M. J., Mettu P. S., Cousins S. W., Tomasi C., Farsiu S. (2015). Retinal Artery-Vein Classification via Topology Estimation. *IEEE Transactions on Medical Imaging*.

[30] Welikala R. A., Foster P. J., Whincup P. H. (2017). Automated arteriole and venule classification using deep learning for retinal images from the UK Biobank cohort. *Computers in Biology and Medicine*.

[31] Staal J., Abràmoff M. D., Niemeijer M., Viergever M. A., van Ginneken B. (2004). Ridge-based vessel segmentation in color images of the retina. *IEEE Transactions on Medical Imaging*.

[32] Xu X., Wang R., Lv P. (2018). Simultaneous arteriole and venule segmentation with domain-specific loss function on a new public database. *Biomedical Optics Express*.

[33] Hoover A., Kouznetsova V., Goldbaum M. (2000). Locating blood vessels in retinal images by piecewise threshold probing of a matched filter response. *IEEE Transactions on Medical Imaging*.

[34] MESSIDOR MESSIDOR; methods for evaluating segmentation and indexing techniques dedicated to retinal ophthalmology. http://messidor.crihan.fr/description-en.php.

[35] Qureshi T. A., Habib M., Hunter A., Al-Diri B. A manually-labeled, artery/vein classified benchmark for the DRIVE dataset.

[36] Carmona E. J., Rincón M., García-Feijoó J., Martínez-de-la-Casa J. M. (2008). Identification of the optic nerve head with genetic algorithms. *Artificial Intelligence in Medicine*.

[37] Kauppi T., Kalesnykiene V., Kamarainen J.-K. (2006). DIARETDB0: Evaluation database and methodology for diabetic retinopathy algorithms. *Machine Vision and Pattern Recognition Research Group*.

[38] Kauppi T., Kalesnykiene V., Kamarainen J.-K. DIARETDB1 diabetic retinopathy database and evaluation protocol.

[39] Giancardo L., Meriaudeau F., Karnowski T. P. (2012). Exudate-based diabetic macular edema detection in fundus images using publicly available datasets. *Medical Image Analysis*.

[40] Al-Diri B., Hunter A., Steel D., Habib M., Hudaib T., Berry S. REVIEW - A reference data set for retinal vessel profiles.

[41] Qureshi T. A., Amin H., Hussain M., Qureshi R. J., Al-Diri B. Automatic localization of the optic disc in retinal fundus images using multiple features.

[42] Tramontan L., Grisan E., Ruggeri A. An improved system for the automatic estimation of the Arteriolarto-Venular diameter Ratio (AVR) in retinal images.

[43] Fraz M. M., Badar M., Malik A. W., Barman S. A. (2018). Computational methods for exudates detection and macular edema estimation in retinal images: a survey. *Archives of Computational Methods in Engineering*.

[44] Fraz M. M., Jahangir W., Zahid S., Hamayun M. M., Barman S. A. (2017). Multiscale segmentation of exudates in retinal images using contextual cues and ensemble classification. *Biomedical Signal Processing and Control*.

[45] Badar M., Shahzad M., Fraz M. Simultaneous segmentation of multiple retinal pathologies using fully convolutional deep neural network.

[46] Abdullah M., Fraz M. M., Barman S. A. (2016). Localization and segmentation of optic disc in retinal images using circular Hough transform and grow-cut algorithm. *PeerJ*.

[47] Basit A., Fraz M. M. (2015). Optic disc detection and boundary extraction in retinal images. *Applied Optics*.

[48] Zahoor M. N., Fraz M. M. (2017). Fast optic disc segmentation in retina using polar transform. *IEEE Access*.

[49] Badrinarayanan V., Kendall A., Cipolla R. (2017). SegNet: A Deep Convolutional Encoder-Decoder Architecture for Image Segmentation. *IEEE Transactions on Pattern Analysis and Machine Intelligence*.

[50] Yan Z., Yang X., Cheng K.-T. (2018). A skeletal similarity metric for quality evaluation of retinal vessel segmentation. *IEEE Transactions on Medical Imaging*.

[51] Yan Z., Yang X., Cheng K.-T. (2018). Joint segment-level and pixel-wise losses for deep learning based retinal vessel segmentation. *IEEE Transactions on Biomedical Engineering*.

[52] Everingham M., van Gool L., Williams C. K. I., Winn J., Zisserman A. (2010). The pascal visual object classes (VOC) challenge. *International Journal of Computer Vision*.

[53] Russakovsky O., Deng J., Su H. (2015). Imagenet large scale visual recognition challenge. *International Journal of Computer Vision*.

[54] Akbar S., Hassan M. T., Akram U., Yasin U. U., Basit I. AVRDB: annotated dataset for vessel segmentation and calculation of arteriovenous ratio.

[55] Jarrett K., Kavukcuoglu K., Ranzato M., LeCun Y. What is the best multi-stage architecture for object recognition?.

[56] Long J., Shelhamer E., Darrell T. Fully convolutional networks for semantic segmentation.

[57] Niemeijer M., Staal J., Van Ginneken B., Loog M., Abràmoff M. D. Comparative study of retinal vessel segmentation methods on a new publicly available database.

[58] Al-Diri B., Hunter A. (September 2009). Automated measurements of retinal bifurcations. *World Congress on Medical Physics and Biomedical Engineering*.

[59] Perez-Rovira A., MacGillivray T., Trucco E. VAMPIRE: vessel assessment and measurement platform for images of the REtina.

[60] Badawi S. A., Fraz M. M. (2018). Optimizing the trainable B-COSFIRE filter for retinal blood vessel segmentation. *PeerJ*.

[61] Anaya J., Barbu A. (2018). RENOIR – A dataset for real low-light image noise reduction. *Journal of Visual Communication and Image Representation*.

